# Micro-Organisms and the Teeth

**Published:** 1888-08

**Authors:** M. Bissell

**Affiliations:** Camden, S. C.


—*Southern Dental Journal.*

---

ARTICLE VIII.

### MICRO-ORGANISMS AND THE TEETH.

BY DR. M. BISSELL, OF CAMDEN, S. C.

(Read before the South Carolina Dental Association, July 1888.)

The injurious effects of micro-organisms on the human system is a subject of decided interest, one worthy of investigation on the part of the dentist as well as the physician and scientist, especially the action of those found in the mouths of all persons; even when the strictest care is used they will be found in the tartar encrusting the teeth.

If the subject possessed any of the elements of sublimity, my hearers might realize the truth of the maxim, that there is but one step from the sublime to the ridiculous when I broach the subject of microbes being active factors in causing caries in the teeth by boring into them.

The vast number of parasites in the mouths whose peculiarities have not been described may afford room and vergi enough to accord to some of them more active work than has been accorded to them.

The mouth is not a very favorable location in which to examine any species of animalcules when fulfilling their destiny; though the habits of some have been noted, and especially a similar species out of the mouth, but existing in a fluid medium.

There have been numerous instances recorded of the injurious effects caused by the tunneling process of several microbes; for instance, in the moluska we find no utensil by which they are able to accomplish their operations. By what means is the delicate pholos, with a shell as thin as paper, and as brittle as a wafer, enabled to excavate deep tunnels in a rock as hard as flint? Many of the species burrow in stone as well as in more penetrable substances.

Some writers contend that the animal in the shell secretes an acid which acts as a corrosive solvent upon the mass. That there is an acid secretion by some minute microscopic creatures is an admitted fact. May not some species possess the means of making an entrance into the teeth, when portions of these organs are in an abnormal condition, either from defective original formation, or from the lack of the deposits of the elements essential for healthy tooth structure?

Why may not the microbes in the mouth be able to excavate the solid bone, when a similar species out of the mouth, but in a fluid element, can make their way into stone piers and utterly destroy the hardest ship timbers? The process may be slow, but time is an element in many operations, and the process of decay in the dental organs in a majority of cases is retarded, though in some cases it is rapid.

There does not appear to be very strenuous objections to the hypothesis as one of the causes of caries in the teeth, because of their usual dense character, or of the infinitesimal character of the animal, when the fact has been established of the destructive powers of the marine animals. The enamel being much more viterous than the dentine, it would require longer time to be affected, and not being as highly organized as the dentine, the ravages of the parasite would not be indicated until the crowns and dentine had become honey-combed by the operation, and the impression would be entertained that the destruction was from the decay having an internal origin.

An instance of the working of one species may be noted in the "tendenavelos," the ship worm. The tunnelings of this animal are so close together that the partitions between them are not thicker than writing paper. May it not be the same with those in the mouth. They may excavate the solid portions of the enamel and dentine, and the teeth having no support but the cartalaginous portions, the teeth are crushed in. It is not improbable but that acids may have a part in the destruction.

As another instance of the destructive effects of these minute organisms, may be given the action of one species; which appears to be capable of destroying the under surface of iron rails. Examinations were made as to the failure of some of the rails, when the top surface appeared perfect; when taken up the under surfaces were found reduced to a red powder and on close examination it was found to have been the work of a minute organism that secreted an acid. A portion of the rail was honey- c mbed, not entirely dissolved

In connection with the above statements, I will quote a passage from Liber and Rottenstein, on caries of the teeth, which has, I think, a direct bearing on the subject. They say: "There is a constitutional difference in the teeth, which renders some much more sensitive to the influences that causes decay than others. These influences are not so much internal and vital, as external and chemical—decay begins at the surface, and there it must be checked, if checked at all. It is caused by acids, and by certain fungii, parasitic in character, found abundantly in all mouths. This fungii obtains its greatest size in the interstices of the teeth.

All acids, the mineral and vegetable, act promptly on the teeth. The acids are taken with food or in the medicine, or are formed in the mouth from acid fermentations of particles of food. But acids alone cannot account for all the phenomena of caries, though they play an important part in making the teeth porous, and when in this porous state, having lost their normal consistency, the fungii (the parasites) penetrate the enamel and dentine, and produce the destructive effects more rapidly than the acids alone." Liber and Rottenstein seem to accord to the microbes decided working powers.

Found as these formations are in all mouths, the solution of the question whether they are absolute factors in causing caries would be an interesting development. The hypothesis as to the active operation of an animalcule being one of the causes of caries, may not come within the investigations of Pasteur and Koch, yet I am not inclined to forego my impressions, believing there are facts affording ample grounds for the opinion, and the statements of Liber and Rottenstein, with others by Merril, an investigator in Berlin, are not wanting as proof of the hypothesis I have ventured to offer.—*Southern Dental Journal.*

---

